# Bionic Sensors for Biometric Acquisition and Monitoring: Challenges and Opportunities

**DOI:** 10.3390/s25133981

**Published:** 2025-06-26

**Authors:** Haoran Yu, Mingqi Ma, Baishun Zhang, Anxin Wang, Gaowei Zhong, Ziyuan Zhou, Chengxin Liu, Chunqing Li, Jingjing Fang, Yanbo He, Donghai Ren, Feifei Deng, Qi Hong, Yunong Zhao, Xiaohui Guo

**Affiliations:** 1 School of Integrated Circuits, Key Laboratory of Intelligent Computing and Signal Processing of Ministry of Education, Anhui University, Hefei 230601, Chinaguoxh@ahu.edu.cn (X.G.); 2 Huaibei Zhongtai Electromechanical Engineering Co. Ltd., Huaibei 235047, China; 3 Huadong Photo-Electron IC Institute, Bengbu 233030, China

**Keywords:** bionic sensors, bioelectric signal sensors, biomarker sensors, biomechanical sensors, multimodal integrated sensors, wearable devices

## Abstract

The development of materials science, artificial intelligence and wearable technology has created both opportunities and challenges for the next generation of bionic sensor technology. Bionic sensors are extensively utilized in the collection and monitoring of human biological signals. Human biological signals refer to the parameters generated inside or outside the human body to transmit information. In a broad sense, they include bioelectrical signals, biomechanical information, biomolecules, and chemical molecules. This paper systematically reviews recent advances in bionic sensors in the field of biometric acquisition and monitoring, focusing on four major technical directions: bioelectric signal sensors (electrocardiograph (ECG), electroencephalograph (EEG), electromyography (EMG)), biomarker sensors (small molecules, large molecules, and complex-state biomarkers), biomechanical sensors, and multimodal integrated sensors. These breakthroughs have driven innovations in medical diagnosis, human–computer interaction, wearable devices, and other fields. This article provides an overview of the above biomimetic sensors and outlines the future development trends in this field.

## 1. Introduction

**Figure 1:**
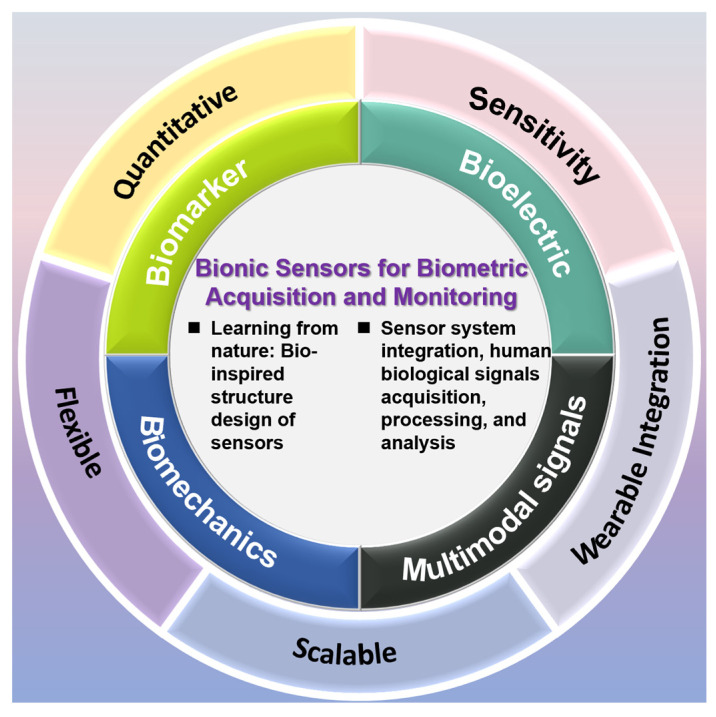
Introduction to sensor types.

With the coordinated development of materials science, artificial intelligence and wearable technology, next-generation bionic sensor technology is embracing significant development opportunities while also facing technical challenges [1,2]. These sensors have extensive application value in human biological signal monitoring and can collect various physiological parameters in real time, including electroencephalogram (EEG) activity, electromyography (EMG) signals, metabolic molecules, specific biomarkers, respiratory waveforms, and body temperature changes. Through a systematic signal processing procedure—including data collection, intelligent analysis and information extraction—these biological signals can be transformed into clinically valuable diagnostic data. This not only provides technical support for health care and precision medicine, but also opens new avenues for health management and basic medical research. Biological sensors originate from human research on information acquisition. As shown in Figure 1, bionic sensors can be classified into the following categories: bioelectric signal sensors (including ECG sensors, EEG sensors, and EMG sensors), molecular sensors (including small molecule biomarker sensors, large molecule biomarker sensors, and complex-state biomarker sensors), biomechanical sensors, and multimodal sensors [3,4,5].

After billions of years of evolution, nature has developed countless exquisite sensing mechanisms. From the nanoscale coloration of butterfly wings to fluid sensing of shark lateral lines, biological sensors often exhibit significant advantages such as high efficiency, low energy consumption, and adaptability. Biomimetic sensor design is achieved by deconstructing these biosensing principles and transforming them into engineering solutions. Currently, this field primarily focuses on three research directions: (1) microstructure imitation, such as wide-angle visual sensors imitating insect compound eyes; (2) development of functional materials, such as superhydrophobic sensing surfaces inspired by the lotus leaf effect; and (3) intelligent system integration, such as the distributed sensing array constructed using neural networks. This interdisciplinary research method not only drives sensor technology towards greater sensitivity and energy efficiency but also provides innovative solutions for challenges like complex environmental monitoring and precision medical diagnosis. In the future, with advancement in biological microscopy technology, computational simulation, and artificial intelligence, more mysteries of nature will be unlocked, leading to groundbreaking sensor design paradigms.

Researchers have drawn inspiration for sensors from nature: the structure of octopus suckers inspired the development of a dual-network hydrogel electrocardiographic sensor; mimicking the structure of starfish combined with motion compensation algorithms to create a sensor capable of measuring multiple electrical signals; mimicking the spider web distribution of electrodes helps in collecting electroencephalogram signals; the bionic ginkgo leaf veins structure promoted sweat directional transport; the Venus flytrap inspired the design of soft robots; the snail’s stalked eye structure inspired a machine structure to achieve 360° rotation; the frog’s leg inspired the development of a mechanical sensor; and inspired by the tactile corpuscle, a tactile particle bionic piezoresistive sensor was developed.

These technologies are supported by research directions such as materials science and biomechanics. New functional materials such as conductive hydrogels and MXene nanosheets endow the sensors with tissue-like mechanical properties and self-healing capabilities, and breakthroughs in manufacturing processes such as 3D printing and the sandpaper template method make it possible to prepare bionic micro-nano structures on a large scale. At present, biomimetic sensors are entering daily life from laboratories, playing a role in fields such as healthcare [1], human–computer interaction [2], food detection [3], and wearable devices [4,5]. This article aims to introduce readers to the field of biomimetic sensors and provide an outlook on the future development trends in this area. Next, this article will introduce sensors from four aspects: bioelectric signal sensors, molecular sensors, biomechanical sensors, and multimodal sensors.

## 2. Bioelectric Signal Sensors

Bionic strategies have enabled bioelectric signal sensors to overcome the limitations of traditional devices in dynamic physiological environments. The core innovation lies in mimicking the structure and function of biological systems: structural bionics (such as the flexible adhesion of octopus suckers and the stress dispersion of spider webs) endow sensors with the ability to conformally adapt to biological tissues, eliminating motion interference; functional bionics (such as the self-repairing properties of skin and the ion conduction mechanism at the nerve–electrode interface) ensure long-term stable signal acquisition; and system bionics (such as the multi-arm coordinated perception of starfish and the electromechanical coupling of muscle–skin) achieve multimodal signal fusion analysis.

Bioelectric or bio-potential signals, as an important electrophysiological indicator reflecting human physiological functions, have extensive application value in the fields of life science and clinical medicine, including multiple aspects such as disease diagnosis, patient monitoring, and health prevention [6,7]. However, due to the weak amplitude of bioelectric signals (typically ranging from microvolts to millivolts) and their susceptibility to environmental electromagnetic interference and electromyographic artifacts and other noises (the amplitude of the interference signal can be dozens of times that of the signal itself), professional bioelectric sensors are required for signal acquisition. Among them, how to achieve reliable application on human skin and stable signal acquisition is the key point to be considered [8,9]. Bionic sensors constructed based on hydrogel-based, fabric-based, and bionic skin-based materials or devices have shown good biocompatibility and have been widely studied and applied in recent years.

Biomimetic electrical signal sensors face three core challenges in practical applications: biologically active materials (such as enzymes and cells) are easily affected by temperature, humidity, and acidity, leading to deactivation; flexible conductive materials (such as polymers) are prone to fatigue aging and signal drift due to long-term use; meanwhile, weak bioelectric signals (such as μV electromyographic signals) are susceptible to electromagnetic fields, power frequency electric fields, or environmental electrostatic interference, especially in multimodal perception where tactile and temperature signals are prone to coupling crosstalk; in addition, the integration process of biomaterials and electronic components is complex (such as the verticality control of nanoscale structures), and the difficulty of large-scale production is high. Moreover, the adaptability to environmental factors such as underwater corrosion and biological pollution is insufficient. For these problems, cutting-edge solutions focus on material and packaging innovation—for example, bionic gel is used to package artificial hair cells to improve corrosion resistance, and self-healing polymer is used to dynamically repair cracks to extend life; in terms of anti-interference, a triple shielding structure (magnetic shielding layer, electromagnetic shielding layer, and drive cable) is used to suppress electromagnetic noise, and adaptive filtering algorithm and AI real-time calibration of temperature drift are integrated; utilizing micro-nano technologies (such as silicon-based cantilever) at the manufacturing end to simplify bio-electronic integration, while integrating piezoelectric/frictional power generation modules (such as PZT ceramics) to achieve a battery-free energy supply system for environmental energy harvesting. The future breakthrough direction will focus on multimodal perception dynamic fusion and biomimetic networking architecture, building an intelligent perception network that combines low power consumption and high robustness.

### 2.1. Electrocardiogram Sensors

The early diagnosis and long-term monitoring of cardiovascular diseases require electrocardiogram (ECG) sensors with exceptional sensitivity, comfort, and environmental adaptability. In recent years, biomimetic ECG sensors have emerged as a superior alternative to traditional non-biomimetic counterparts [6,7,8,9]. By emulating biological structures and functions—such as skin-like flexibility, self-healing capabilities, and biocompatible interfaces—these innovative sensors outperform conventional models in signal quality, wearing comfort, and dynamic anti-interference performance.

Traditional ECG sensors face significant practical limitations. As documented in [6], conventional optical ECG monitors suffer from low sensitivity and often fail to detect low-amplitude waves like U and J waves, which are crucial for comprehensive cardiac analysis. Research in [7] highlights that “wet sensors” such as Ag/AgCl electrodes degrade signal quality over time, require laborious skin pretreatment, and demand frequent replacement of conductive gels, compromising user convenience. Moreover, traditional rigid designs are prone to motion artifacts during sleep or daily activities [8], and their poor ergonomics lead to discomfort during extended use. Existing dynamic ECG monitors, being bulky medical devices, necessitate professional electrode placement and post-hoc analysis [9], limiting real-time, personalized health tracking.

**Figure 2:**
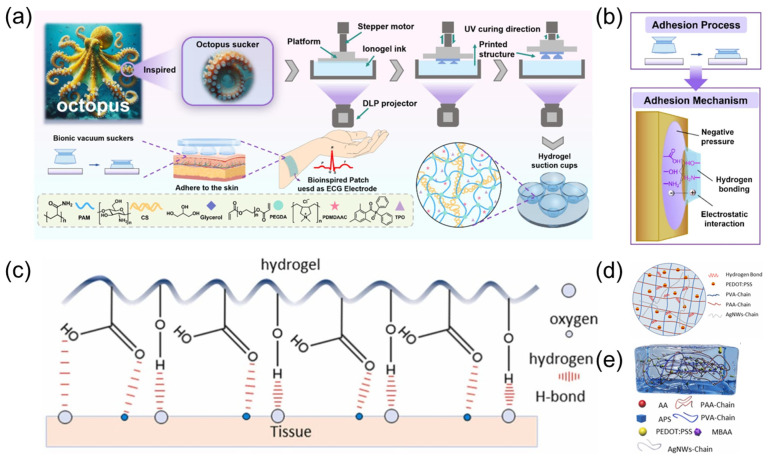
(**a**) Schematic illustration showing the preparation process of the PCP hydrogel. Reproduced with permission from ref. [10] with permission from Elsevier. (**b**) The pressing technology, adhesion design and adhesion mechanism of octopus sucker hydrogel model. Reproduced with permission from ref. [10] with permission from Elsevier. (**c**) Principle of adhesion of conductive hydrogels. Reproduced with permission from ref. [11] with permission from Elsevier. (**d**) Structure distribution of double network conductive hydrogel. Reproduced with permission from ref. [11] with permission from Elsevier. (**e**) Schematic diagram of the internal structure of the hydrogel, with the compositional elements of the hydrogel underneath the diagram. Reproduced with permission from ref. [11] with permission from Elsevier.

The bionic hydrogel ECG sensor achieves both high sensitivity and high-comfort dynamic monitoring through material design and structural biomimetics. As shown in Figure 2a, inspired by the octopus sucker structure Lian, et al. [10] developed a DLP 3D-printed PAM/CS double-network hydrogel with dimensionally programmable suction cup arrays for high-performance ECG sensors. The mechanism of octopus suction cups forming edge seals through muscle contraction to generate negative pressure adsorption has been innovatively applied to the hydrogel design (Figure 2b), enabling efficient interfacial sealing and stable adhesion. The concave cavity microstructures of the bio-inspired suction cup form annular sealing regions under compression, with maximum adhesion strength reaching 23.6 kPa. This represents a 3–5-fold enhancement over unstructured hydrogels due to the synergistic effects of negative pressure mechanisms and hydrogen bonding interactions. Nan et al. [11] proposed a wearable ECG monitoring system based on an easily detachable, highly conductive hydrogel. As displayed in Figure 2c–e, the system utilizes polyacrylic acid (PAA) and polyvinyl alcohol (PVA) to construct a dual-network hydrogel matrix, with silver nanowires (AgNWs) and poly(3,4-ethylenedioxythiophene):poly(styrene sulfonate) (PEDOT:PSS) incorporated as conductive fillers, achieving a low contact impedance of 190.79 Ω and 300% strain at break. The mechanical properties are enhanced through hydrogen bonding interactions within the hydrogel. The system integrates a flexible printed circuit board (FPCB) for ECG acquisition and wireless transmission, enabling stable eight-hour continuous monitoring. Combined with a lightweight convolutional neural network (CNN) algorithm, it achieves 97.9% accuracy in classifying ECG features across lying, sitting, and standing postures.

**Figure 3:**
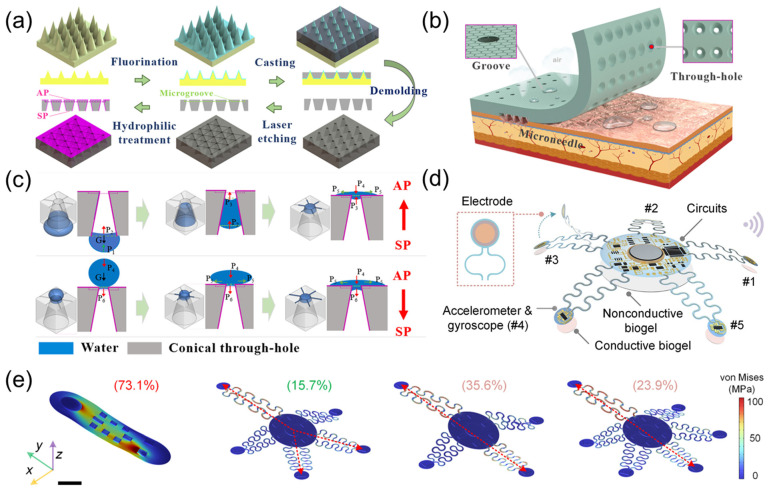
(**a**) Schematic diagrams of the fabrication process of the adhesion material with the conical through-hole and hexagonal microgroove. Reproduced with permission from ref. [12] with permission from ACS. (**b**) Schematic illustration of bionic inspiration for the design of the patch that can be stably adhered to skin for bioelectrical signal monitoring in health application. The conical through-hole (Laplace pressure difference) and hexagonal microgroove (capillary force) are used to directionally transport sweat from skin to the air environment, and the multi-mechanism adhesion of the adhesive material and microneedle array enhances the adhesion capability on human skin. Reproduced with permission from ref. [12] with permission from ACS. (**c**) Schematic illustrations of the water transport mechanism on the SP-surface and AP-surface by the conical through-hole and hexagonal microgroove. Reproduced with permission from ref. [12] with permission from ACS. (**d**) Schematic diagram of a starfish-shaped device used for three-mode cardiac monitoring during exercise. Reproduced with permission from ref. [13] with permission from AAAS. (**e**) FEA simulations of stress distribution under a 10 mm displacement applied to top-left corners of various soft bioelectronic device configurations, including a traditional monolithic design and starfish-inspired designs with four, five, and six arms. The most pronounced stress coupling occurs along the diagonal axes in all configurations, with the five-arm configuration exhibiting the lowest stress coupling coefficient at 15.7%, significantly lower than four-arm (35.6%), six-arm (23.9%), and monolithic (73.1%) designs. Color bar, stress distribution. Scale bar, 10 mm. Reproduced with permission from ref. [13] with permission from AAAS.

Zhang et al. [12] drew inspiration from the gecko toe bristles, waterfowl drinking water, and hexagonal microgrooves to develop a biomimetic wicking-breathable patch for long-term stable monitoring of bioelectric signals (Figure 3a). As illustrated in Figure 3b, the patch can be stably adhered to the skin, enabling biopotential signal monitoring for health applications. The patch utilizes tapered through-holes (Laplace differential pressure) and hexagonal microgrooves (capillary force) to directionally transport sweat from the skin to the air environment, and the multi-mechanism bonding of adhesive materials and microneedle arrays enhances adhesion to human skin. The patch adopts an Ag/Ni microneedle array, which can penetrate the epidermis painlessly to collect electrocardiogram signals. Combined with biomimetic PDMS-t adhesion material, as displayed in Figure 3c, the conical through-hole and hexagonal microgrooves of the patch discharge sweat in a directed manner through Laplace pressure difference and capillary effect, ensuring skin comfort and high breathability. Under 2 N preload, the dry and wet adhesion of the patch reached 2.13 N and 1.85 N, respectively, which was significantly better than the traditional gel patch. Experiments have shown that the patch can clearly capture the P-wave and T-wave characteristics of ECG on both dry and wet skin, especially in humid environments where signal recognition is superior to conventional products.

As illustrated in Figure 3d, inspired by the pentaradial symmetry of starfish, Chen et al. [13] developed an innovative wearable bioelectronic system for high-fidelity cardiac signal monitoring and real-time disease diagnosis in dynamic environments. The system adopts a pentaradial configuration with five flexible sensing arms, reduces the stress coupling coefficient to 15.7% through mechanically coupling design (Figure 3e), and integrates an accelerometer gyroscope unit and a gold electrode to achieve synchronous acquisition of electrocardiogram signals and cardiac mechanical signals. By using signal compensation algorithms and a transformer model, a 35 dB signal-to-noise ratio (SNR) can be maintained during intense activities such as running, accurately capturing key parameters such as QT interval and heart rate variability. The real-time diagnostic accuracy of the machine learning model based on trimodal signal fusion for atrial fibrillation, myocardial infarction, and heart failure reached 91.31–94.03%, which was more than 50% higher than that of single signal diagnosis. At the same time, the device weighs only 1.7 g.

### 2.2. Electroencephalogram Sensors

The EEG sensor is a key device for monitoring neural signals by detecting electrical activity on the scalp or in the cerebral cortex. Its core principle is based on the weak potential changes generated by neuronal discharges. The current EEG sensors are mainly divided into three categories: invasive, semi-invasive, and non-invasive. Non-invasive EEG sensors [14,15,16] collect signals through scalp contact. Semi-invasive EEG sensors [17] require surgical implantation in the dura mater between the skull and cerebral cortex. Invasive EEG sensors [18] are directly implanted into brain tissue for high-precision medical scenarios, such as epilepsy lesion localization. In recent years, advances in flexible electronics have led to the development of flexible electrodes that conform to the scalp. Combined with wireless transmission technology, these innovations have driven EEG devices toward lightweight design and long-term monitoring. Inspired by the bionic structure of spider webs, the issue of poor signal quality in traditional EEG sensor networks has been solved. This improvement also makes the technology suitable for infants with curly or tightly coiled hair.

**Figure 4:**
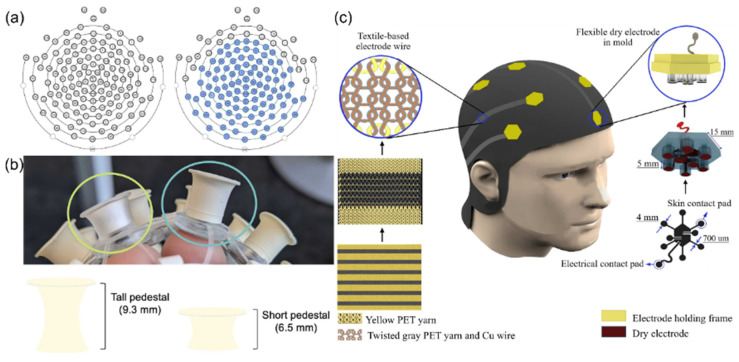
(**a**) The image on the left depicts the standard, or short, 128-channel infant net, which contains 128 electrodes and pedestals that are all 6.5 mm tall and made with a soft plastic material. The image on the right depicts the modified tall net, where the electrodes shaded in blue are 9.3 mm tall and made of a more rigid plastic material. Reproduced with permission from ref. [19] with permission from Elsevier. (**b**) Circled in green (left) is an example of the standard, or short, electrodes of 6.5 mm height from the infant net which uses a clear soft pedestal. Circled in blue (right) is an example of the modified, tall, electrodes of 9.3 mm height and more rigid molded rubber material. Reproduced with permission from ref. [19] with permission from Elsevier. (**c**) Schematic illustration of the clutter-free e-textile EEG cap. Right: The steps of fabricating a flexible electrode. Left: The steps of fabricating textile-based electrode wires. Reproduced with permission from ref. [20] with permission from Elsevier.

The structure of EEG caps is inspired by the extensibility of spider webs and biological tissues. Mlandu et al. [19] developed a Magstim EGI 128-channel HydroCel Geodesic Sensor Net with saline-based system and evaluated its performance. This study compared the standard short sensor network with 6.5 mm pedestals to an improved tall sensor net with partially 9.3 mm tall electrode pedestals (Figure 4a,b), showing that the improved tall net significantly reduced the impedance of the online reference electrode and the average electrode, especially for infants with hair length exceeding 1 cm. The modified tall sensor net design allowed the tall net to fit more effectively on complex scalp surfaces, reducing the impact of hair interference on the signal. Experiments confirmed that using taller rigid plastic-encased pedestals enhanced the stability of sensor contact with the scalp, providing a more reliable EEG signal acquisition solution for infants with tightly coiled hair.

The use of horseshoe-shaped wiring and flexible polymers such as polyimide (PI) simulates the extensibility of biological tissues while making the overall structure less prone to metal fatigue. Kourosh Motiepor et al. [20] fabricated a bionic stretchable electronic textile EEG cap (Figure 4c), combining polyimide-supported dry electrodes with textile-based electrode wires to solve the problem of traditional EEG equipment being rigid and uncomfortable. The design mimics the scalp surface characteristics, achieving flexible contact with the scalp through a silicone elastomer-embedded nickel-based electrode. The horseshoe-shaped knitted conductive yarns were used to simulate the extensibility of biological tissues to construct a double-layer weft-knitted structure, achieving stretchable wiring of electrode leads. Static and dynamic tensile testing results indicated that this electronics exhibits a maximum stretch rate of 110% for the leads and long-term stability (3200 cycles of 40% deformation). Electrode–skin impedance tests proved that its impedance is comparable to that of commercial wet electrodes. This integrated design eliminates messy wires, achieving breathable wearable structures through textile technology, providing a bionic solution for long-term EEG monitoring.

**Figure 5:**
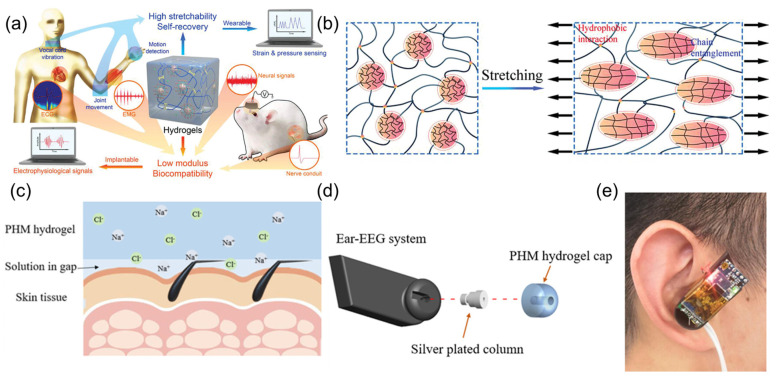
(**a**) Schematic diagram of the hydrogel structure and its applications as wearable and implantable sensors. Reproduced with permission from ref. [21] with permission from Wiley. (**b**) The schematic deformation of microgels in hydrogel networks by stretching. Reproduced with permission from ref. [21] with permission from Wiley. (**c**) Schematic diagram of the PHM hydrogel used in bioelectrode. Reproduced with permission from ref. [22] with permission from ACS. (**d**) Structural diagram of the wireless ear-EEG system. Reproduced with permission from ref. [22] with permission from ACS. (**e**) Wearing diagram of the wireless ear-EEG system. Reproduced with permission from ref. [22] with permission from ACS.

Metal electrodes implanted in the brain are encapsulated by glial cells after a certain period, interfering with sensing results. As demonstrated in Figure 5a, a high-performance hydrogel sensor based on microgel crosslinking was prepared [21]. Addressing the issues of foreign body reactions and signal attenuation caused by the chemical and mechanical property mismatch between traditional implantable sensors and neural tissue, the research team innovatively used poly-N-isopropylacrylamide microgels as large-sized crosslinking centers, combined with hydrophobic association to construct a dynamic network structure. The fabricated HM-2 hydrogel material showed low modulus, high extensibility, and excellent fatigue resistance. The hydrogel maintained stable performance in 200 cycles of 500% strain stretching and 500 compression tests (Figure 5b), and its elastic modulus was highly matched with that of neural tissue. Experiments showed that HM-2, as a wearable sensor, could accurately monitor physiological signals such as joint movement, voice vibration, electrocardiogram, and myoelectricity. After implantation in the hippocampal CA1 region of rats, it could stably record the local field potential for up to 4 weeks, with an attenuation rate of only 0.6%, while the attenuation rate of the platinum electrode is 34.7%. Comparative studies found that the HM-2 hydrogel material reduced glial cell aggregation by over 60%. Additionally, the neural conduit prepared by this hydrogel could effectively bridge sciatic nerve defects, transmit compound muscle action potentials, maintain signal stability for over 28 days, and has good biocompatibility. Ge et al. [22] proposed a high-conductivity, low-impedance ionic conductive hydrogel material specifically for ear-EEG signal acquisition. The material, prepared by introducing hydroxypropyl methyl cellulose (HPMC) into a poly (vinyl alcohol) (PVA) matrix to form a physically cross-linked hydrogel structure (Figure 5c), exhibited an electrical conductivity of 7.26 S/m and impedance below 8 Ω after immersion in 5 M NaCl solution. Its Young’s modulus (5.394 kPa) exhibited good biological adaptability to human skin and could have conformal contact with the ear canal to achieve stable signal detection (Figure 5d,e). The PHM hydrogel bioelectrode showed excellent biocompatibility, causing no obvious skin irritation even after 8 h of continuous wear. Based on this material, the researchers constructed a wireless ear-EEG acquisition system for real-time extraction of attention feature values through β-band frequency analysis, which can achieve a six-level LED color feedback mechanism. The experiment verified the effectiveness of the system in monitoring attention fluctuations caused by acoustic interference. Compared with carcinogenic carbon nanotube-based electrodes and rigid gold-tip electrodes, this material demonstrated high biological adaptability and wearing comfort through Staphylococcus aureus inhibition testing and long-term wear experiments.

### 2.3. Electromyography Sensors

Nowadays, electromyography sensors have been developed and are widely used in human–computer interaction [23,24], medical rehabilitation [25,26], sports analysis [27,28], and other fields.

**Figure 6:**
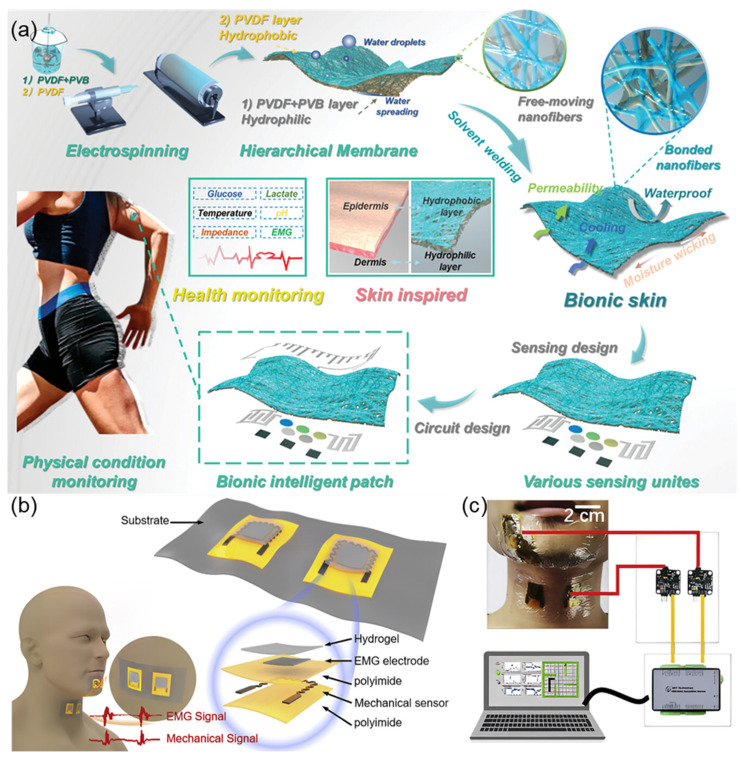
(**a**) The schematic diagram of bionic intelligent skin with air permeability, moisture permeability, waterproofness, multifunctional sensing property, and big data analysis for different physical conditions. Reproduced with permission from ref. [29] with permission from Wiley. (**b**) DGEMS with key components labeled. Each patch contains two DGEMS. Two patches are placed on the chin and throat to collect bio-signals simultaneously, and the corresponding waveforms are shown in the bottom graph. Reproduced with permission from ref. [30] with permission from Elsevier. (**c**) Schematic showing the system consisting of two patches, a data acquisition card, and a computer. Reproduced with permission from ref. [30] with permission from Elsevier.

Shi et al. [29] fabricated an intelligent bionic skin patch featuring hierarchical bionic secondary structures fabricated via controlled electrospinning technology (Figure 6a). The patch exhibits human skin-like breathability (3.3 mL/s air permeability) and moisture management (16.5 kg m^−2^ d^−1^ WVTR) with unidirectional sweat-wicking functionality through its Janus architecture (hydrophilic PVB/PVA-Cu inner layer and hydrophobic PVDF outer layer). Mechanical stability was enhanced through solvent welding technology, achieving skin-matched tensile strength (20.7 MPa) and elongation (108%). The system integrates GaInSn liquid metal (LM)-based flexible circuits with Prussian blue-modified electrochemical sensors for in situ multiplex detection of sweat metabolites (glucose: 3.6 nA μM^−1^ sensitivity; lactate: 156.6 nA mM^−1^ sensitivity) and electrophysiological monitoring (EMG signals with 10.7 dB SNR). This comprehensive sensing platform enables personalized health management and rehabilitation engineering applications through real-time physiological big data analysis. Inspired by the biological mechanism of speech generation, Tian et al. [30] proposed a bio-inspired dual-channel graphene-based electromyographic and mechanical sensor (DGEMS) system. Figure 6b,c illustrate the working principle of the system, which improves the performance of speech recognition by simultaneously collecting EMG signals and mechanical signals. The system adopts a dual biological channels design, analyzing the generation mechanism of human speech through the synergistic effect of muscle electrical activity (captured by graphene-based EMG electrodes) and skin surface deformations (monitored by graphene-based mechanical sensors). This data fusion model overcomes the limitations of traditional single-signal sources, maintaining over 95% recognition accuracy in noisy environments, with 100% accuracy achieved on the digits dataset. The bio-inspired architecture effectively decreases the number of electrodes, providing new ideas for wearable voice interaction devices.

## 3. Biomarker Sensors

Biomolecular sensors, through biomimetic recognition and microfluidic technology, have achieved precise monitoring of ultra-low concentration biomarkers: biomimetic molecular recognition (such as enzyme–substrate lock-and-key mechanism simulation, antibody–antigen binding biomimicry) endows sensors with high specificity, significantly enhancing detection sensitivity; microfluidic structure biomimicry (such as directional transport in ginkgo leaf veins, capillary adsorption in tree frog toe pads) enables efficient collection and separation of microliter biological fluids; multi-target collaborative analysis (inspired by metabolic networks in the body) supports simultaneous detection of glucose, lactic acid, and proteins in sweat/blood.

The main difficulties faced by biomimetic molecular signal sensors include insufficient biocompatibility and stability (traditional materials are easily degraded or cause rejection reactions in biological environments), low signal conversion efficiency (the conversion of molecular recognition events to electrical signals is susceptible to environmental noise interference, and long-term use is prone to signal drift), limited selectivity (similar molecules in complex samples are prone to cross reactions), and integration challenges (miniaturization and power control of multimodal sensing are difficult). These issues may be addressed through the development of novel nanomaterials (such as graphene, nanoporous gold, MOFs) that enhance biocompatibility and sensitivity through high specific surface area and biomimetic modifications; biomimetic multilevel structure design (such as combining three-dimensional nanopores, hyperbranched PEG antifouling coatings, and adapter switches) enhances anti-interference ability and prolongs in vivo stability; introducing microfluidic technology to achieve precise sample manipulation and reduce contamination; combining artificial intelligence algorithms (such as neural networks) to optimize signal processing and improve selectivity and noise resistance performance; and designing biodegradable devices and self-powered systems to reduce implantation risks and solve energy problems.

### 3.1. Small Molecule Marker Sensors

Glucose sensors are primarily classified into three major types based on their sensing principles: electrochemical, optical, and semiconductor sensors. Electrochemical sensors [31,32,33,34] dominate the field, generating electrical signals via catalytic reactions mediated by glucose oxidase or dehydrogenase, though non-enzymatic approaches have also been explored [35]. Optical sensors [36,37] detect glucose by changes in fluorescence intensity, surface plasmon resonance, or photonic crystal structures, offering advantages against electromagnetic interference but are less portable. Semiconductor sensors [38] use nanomaterials (such as graphene, carbon nanotubes) to modify electrodes, reflecting concentration information through conductivity changes, and have recently achieved rapid response by integrating with microfluidic technology. Although various sensors are widely used in medical monitoring (such as continuous glucose monitors), the food industry, and bioreactor control, traditional enzyme-based sensors still face challenges such as enzyme activity loss and limited detection environments. This has prompted researchers to turn to bionic strategies—building highly stable detection systems through artificial enzyme simulation, molecular imprinting, or DNA aptamer technology, opening new avenues for next-generation glucose sensing.

**Figure 7:**
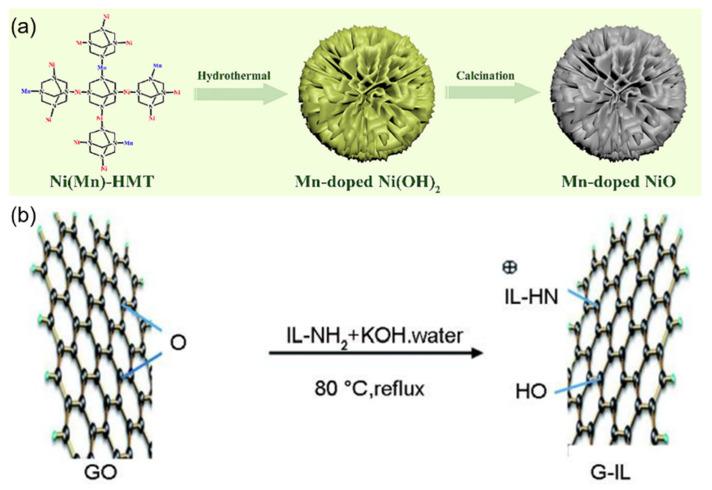
(**a**) Schematic diagram of the synthesis of silver ear-like Mn-NiO nanomaterials. Reproduced with permission from ref. [39] with permission from Elsevier. (**b**) Schematic diagram of the connection between ionic liquid and graphene. Reproduced with permission from ref. [40] with permission from Elsevier.

As illustrated in Figure 7a, the hydrothermal-calcination synthesis yields Mn-doped NiO nanomaterials with unique silver ear-like morphology, which provides optimal surface geometry for glucose oxidation reactions. Gao et al. [39] prepared a nanomaterial of tremella-like Mn-doped NiO through a hydrothermal process followed by calcination, using it as a biomimetic catalyst, which was applied for enzyme-free electrochemical glucose sensing to achieve highly sensitive detection of glucose in human serum. The sensor utilized the enhanced electrocatalytic activity of Mn-doped NiO toward glucose, benefiting from the synergistic effect of the tremella-like nanostructure and Mn doping. The glucose sensor based on Mn-NiO exhibited fast response time (<5 s), high sensitivity (3212.52 μA·mM^−1^·cm^−2^), and long-term stability. This indicates that Mn doping and the tremella-like nanostructure jointly played a crucial role in improving the sensor’s performance. By developing this biomimetic sensor, a new approach is provided for constructing highly sensitive, stable, and low-cost enzyme-free glucose sensors.

Figure 7b demonstrates the molecular bridging mechanism in G-IL/CNTs composites, where ionic liquid molecules form stable connections between graphene sheets and carbon nanotubes, facilitating efficient charge transfer. Zou et al. [40] prepared a glucose biosensor based on 1-methyl imidazole ionic liquid-functionalized graphene/carbon nanotube (G-IL/CNTs) composites, focusing on the synergistic mechanism of the horseradish peroxidase (HRP) and glucose oxidase (GOD) bi-enzyme system. By co-immobilizing HRP and GOD on the surface of the three-dimensional G-IL/CNTs composite, a detection system mimicking the biological cascade reaction was constructed: GOD catalyzed glucose oxidation to produce hydrogen peroxide, which was subsequently reduced through HRP catalysis, forming a complete electron transfer chain. This design combined graphene’s large specific surface area with CNTs’ electron wiring function, achieving efficient electron transfer between enzyme active centers and the electrode interface. The biosensor exhibited excellent electrocatalytic performance with a detection limit of 3.99 × 10^−7^ mol/L, significantly improved sensitivity (53.89 μA mmol^−1^ L cm^−2^), and good stability. This work demonstrates the advantages of integrating nanomaterials with biological enzymes for developing high-performance biosensors.

Lactate sensors, as important tools for biomedical detection and exercise physiology monitoring, focus on improving sensitivity, selectivity, and real-time detection capabilities. The current mainstream sensors can be divided into three categories: electrochemical, optical, and biosensors. Electrochemical sensors [41,42] are centered on enzyme electrode technology, catalyzing lactic acid to produce hydrogen peroxide through lactate oxidase (LOx) and detecting the signal via amperometry or potentiometry, with research also exploring enzyme-free [43] approaches. Optical sensors [44] utilize fluorescent probes or surface plasmon resonance (SPR) technology to convert lactate concentration into changes in optical signals. Biosensors [45] integrate microorganisms, cells, or tissues as biological recognition elements, generating electrical/optical signals through metabolic processes, although they face challenges in preserving live materials despite their high biocompatibility. In recent years, the modification of electrodes with nanomaterials (such as graphene and carbon nanotubes) has significantly improved the detection limits of electrochemical sensors, and molecularly imprinted polymers (MIPs) that mimic the selective recognition mechanism of GLUT proteins on cell membranes have enhanced the stability of sensors as enzyme substitutes.

**Figure 8:**
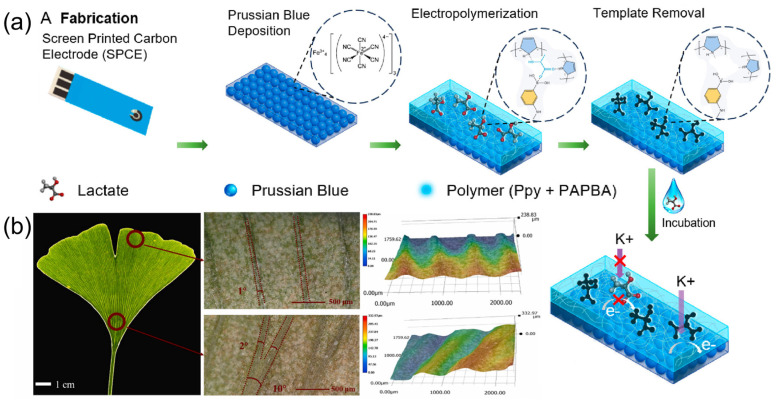
(**a**) The schematic of the fabrication process of eMIP/PB/SPCE-based sensor. Reproduced with permission from ref. [46] with permission from ACS. (**b**) Super-depth-of-field microscope images (SDM) of Ginkgo biloba leaf veins. Reproduced with permission from ref. [47] with permission from Elsevier.

Dykstra et al. [46] proposed a reagent-free electrochemical biosensor based on Prussian blue (PB) and electropolymerized molecularly imprinted polymers (eMIP) for rapid lactate detection in sweat. As illustrated in Figure 8a, the fabrication process involved electrodepositing PB nanoparticles onto screen-printed carbon electrodes (SPCE) as an internal redox probe, combined with an eMIP layer functionalized with 3-aminophenylboronic acid (3-APBA) and pyrrole, achieving high selectivity. The study used electrochemical-surface plasmon resonance (EC-SPR) to validate the template elution and lactate rebinding processes in real time and evaluated the sensor performance via differential pulsed voltammetry (DPV) and electrochemical impedance spectroscopy (EIS). Experimental results indicated that this sensor showed a detection range of 1 to 35 mM with a limit of detection (LOD) of 0.20 mM (signal-to-noise ratio = 3) in 0.1 M KCl and artificial sweat (AS, pH 6.5), along with excellent selectivity against interferents (e.g., urea, glucose). The sensor exhibited reproducibility across 5 regeneration cycles and retained performance after 10 months of ambient storage.

Liao et al. [47] fabricated a wearable epidermal sensor patch with high sweat collection efficiency based on the biomimetic design of Ginkgo biloba leaf veins. Figure 8b displays the super-depth-of-field microscope images (SDM) of these Ginkgo biloba leaf veins, and the research team thoroughly analyzed their wedge-shaped structural characteristics: the veins gradually widen asymmetrically from the leaf margin to the petiole and then drive liquid unidirectional transport through surface curvature differences. The data for this asymmetrical structure show a width increase from 50 ± 9 μm to 250 ± 15 μm and an angle increase from 1 ± 1° to 10 ± 2°. Mimicking this structure, the team innovatively designed a 5° wedge-shaped microfluidic channel with a flow rate of 1.6 ± 0.05 mm/s, which is 40% higher than that of parallel channels (0°). This structure, combined with the burst pressure (BP) regulation mechanism, uses channel width and diverging angle (β) parameters to achieve unidirectional flow of 6 μL sweat, effectively preventing the mixing of old and new sweat. The bionic channel, through the optimization of capillary force (F) and hydrodynamics expressed as F = γcosθP/A, was verified for its transmission efficiency advantage in COMSOL Multiphysics (Version 6.0) simulations, with the liquid passing time reduced by 40%. The study further revealed the regulation mechanism of the wedge structure on the distribution of liquid surface tension: a positive capillary driving force is formed in the direction of contraction, while the direction of expansion is hindered by BP = −2σ[(cosθ_1_*)/b + (cosθ_a_)/h], mimicking the intelligent liquid transport characteristics of Ginkgo biloba veins. This biomimetic design breaks through the structural limitations of conventional microfluidic materials, enabling time-sequence collection at a low sweat secretion rate of 0.4–1.0 μL·min^−1^·cm^−2^, providing a nature-inspired solution for continuous monitoring of trace sweat in wearable devices.

### 3.2. Macromolecular Marker Sensors

Protein biosensors are analytical tools based on biomolecular recognition principles, which achieve high-sensitivity detection of target substances through signal conversion mechanisms. Their core lies in utilizing recognition elements such as antibodies, enzymes, aptamers, or molecularly imprinted polymers to bind with specific proteins with high affinity [48,49,50,51,52]. In recent years, with breakthroughs in nanomaterials, bioengineering, and microfluidic technologies, protein sensors have achieved leapfrog developments in sensitivity, selectivity, and detection throughput, becoming a core technological support in fields such as disease diagnosis, drug development, environmental monitoring, and food safety.

**Figure 9:**
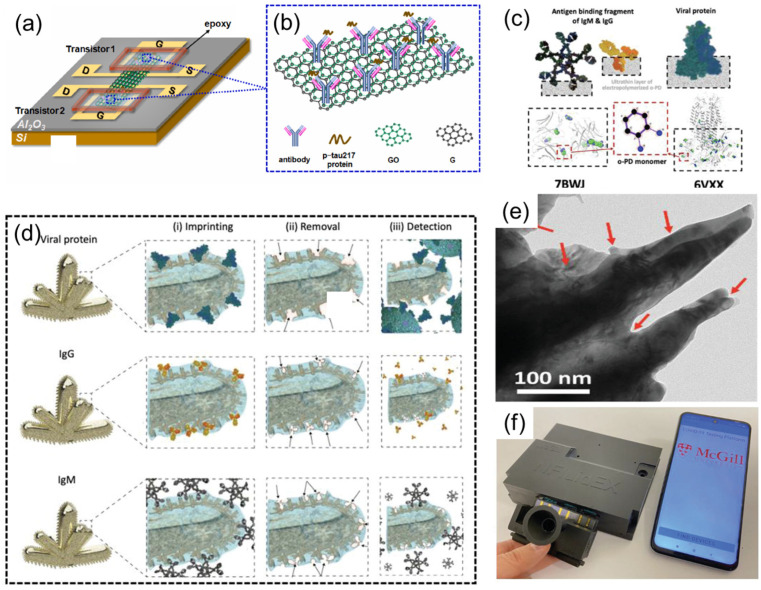
(**a**) A GFET-based biosensor featuring a GO/G layered complex. Reproduced with permission from ref. [53] with permission from Elsevier. (**b**) p-tau217 protein bound with antibody immobilized on GO/G. Reproduced with permission from ref. [53] with permission from Elsevier. (**c**) Molecular docking study of the interaction between o-PD monomers and template protein amino acids, showing the regions of high binding affinity. Reproduced with permission from ref. [53] with permission from Elsevier. (**d**) The formation of rapidly adaptable MIPs templates using viral proteins (top), IgG antibody (middle), and IgM antibody (bottom) for (i) imprinting the polymeric thin film on the nanorough surface of NMI electrodes and (ii) their removal to form the empty geometrical shapes in polymer (iii) for selective detection. Reproduced with permission from ref. [53] with permission from Elsevier. (**e**) Transmission electron microscopy image of the biomimetic NMIs/MIPs. Reproduced with permission from ref. [53] with permission from Elsevier. (**f**) Real image of the NFluidEX quantitative diagnosis and serology testing. Reproduced with permission from ref. [54] with permission from Wiley.

As shown in Figure 9a, Ciou et al. [53] developed a solution-gated field-effect transistor (SGFET) biosensor based on a graphene oxide/graphene (GO/G) layered composite for label-free detection of phosphorylated tau-217 protein (p-tau217), a critical biomarker for Alzheimer’s disease (AD). Figure 9b shows the p-tau217 protein bound with antibody immobilized on the GO/G surface. The team prepared bilayer graphene (BG) via chemical vapor deposition (CVD) and utilized a low-damage plasma treatment (LDPT) to achieve atomic layer oxidation, forming a composite structure with functionalized graphene oxide (containing carboxyl groups) as the top layer and pristine graphene as the bottom layer. Molecular docking studies in Figure 9c reveal the interaction between o-PD monomers and template protein amino acids, showing regions of high binding affinity. The GO surface was covalently immobilized with anti-p-tau217 antibodies for specific recognition, while the underlying graphene transduced electrical signals via π–π interactions, addressing the instability of conventional noncovalent graphene functionalization. Experiments demonstrated a linear detection range for p-tau217 in phosphate-buffered saline (PBS) from 10 fg/mL to 100 pg/mL, with a sensitivity of 18.6 mV/decade (R^2^ = 0.991). The biosensor retained ~90% sensitivity (16.7 mV/decade) in human serum albumin (HSA), achieving a limit of detection (LOD) of 10 fg/mL. Antibody-modified devices exhibited <2% performance variation over 7 days. Hall measurements confirmed that the positively charged p-tau217 induced p-doping via Coulombic interactions, shifting the charge neutrality point (VCNP). This technology addresses the challenge of trace p-tau217 detection in blood, offering a highly specific and stable point-of-care (POC) solution for early AD diagnosis.

Roozbeh Siavash Moakhar et al. [54] developed a multifunctional biosensor named NFluidEX, enabling rapid quantitative detection of respiratory virus infections and immune responses in saliva and blood. The device uses the molecularly imprinted polymer (MIP) template (Figure 9d), gold nanoscale electrode arrays, and microfluidic chip technology (Figure 9e), capable of detecting viral proteins (such as SARS-CoV-2 spike proteins and their Alpha, Delta, Omicron variants) in saliva and RBD IgG/IgM antibodies in blood within 11 min. The study optimized the accuracy of the molecular imprinting template through the protrusion structure of the gold nanoscale electrodes, combined with impedance spectroscopy detection technology, achieving a viral detection limit of 5.89–8.13 pg/mL and an antibody detection limit of 3–7 pg/μL in untreated samples. Clinical tests showed sensitivity and specificity both reaching 100%, highly consistent with RT-qPCR and ELISA results. The device is equipped with a saliva pretreatment module and a smartphone data analysis system, serving as a home testing tool, providing new solutions for early infection diagnosis, immune monitoring, and treatment effect evaluation (Figure 9f).

### 3.3. Complex-State Biomarkers Sensors

**Figure 10:**
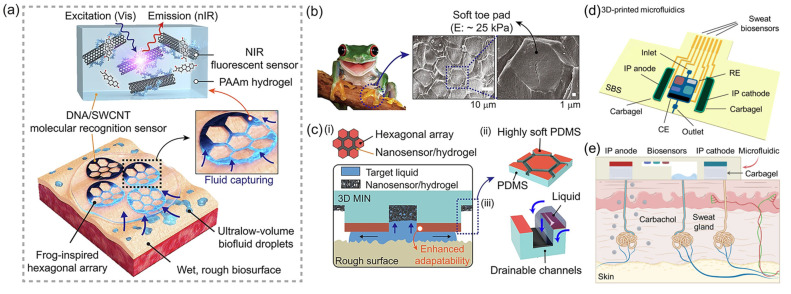
(**a**) Schematic illustration of the adhesion and molecular sensing mechanism of 3D MIN (3D microstructured patch integrated with optical nanosensors) on rough and wet human skin. Reproduced with permission from ref. [56] with permission from RELX Group plc. (**b**) Three-dimensional microstructures observed in the toe pads of a frog (Rhacophorus pardalis). The hexagonal toe pads of the frog are covered with a soft layer of modulus around 25 kPa. The image in (**b**) is adapted from © iStock.com (Asset ID: 1049028724), used under royalty-free license and reproduced with permission from ref. [56] with permission from RELX Group plc. (**c**) Schematic: (i) 3D MIN on wet and rough surfaces, (ii) highly soft polydimethylsiloxane (PDMS) coated hexagonal array, and (iii) drainage and biofluid capture effects facilitated by hexagonal channel structures. Reproduced with permission from ref. [56] with permission from RELX Group plc. (**d**) Schematic illustration of the SSE-based 3D-printed microfluidics. IP, iontophoresis; RE, reference electrode; CE, counter electrode. Reproduced with permission from ref. [55] with permission from AAAS. (**e**) Schematic of the microfluidics-based localized iontophoretic sweat induction through transdermal delivery of muscarinic agent carbachol. Reproduced with permission from ref. [55] with permission from AAAS.

Yeon Soo Lee et al. [55] developed a bio-inspired 3D microstructured optical monolithic patch sensor (3D MIN) inspired by tree frog toe pads (Figure 10a), designed for real-time spatiotemporal molecular tracing of ultralow-volume biofluids. The biological inspiration is shown in Figure 10b, demonstrating the hexagonal toe pad structure. The patch features soft hexagonally aligned PDMS pillars and drainable microchannels, with detailed functional components (Figure 10c), enabling conformal adhesion to rough, dynamic biosurfaces and rapid collection of fluids as low as 0.1 μL/min·cm^2^ through capillary-driven drainage. Embedded within the patch are near-infrared (nIR) fluorescent DNA/SWCNT nanosensors utilizing corona phase molecular recognition (CoPhMoRe), combined with polyacrylamide (PAAm) hydrogel for simultaneous fluid capture and detection. By optimizing elastomeric s-PDMS microarchitecture and Fickian diffusion-based water management, the patch achieves stable wet adhesion (11.3 kPa) and collects ≥75 nL of sweat within 45 s, enabling stand-off nIR camera-based remote analysis without exercise or iontophoresis. The 3D MIN multiarray monitors biomarkers like vitamins B2 (riboflavin), B6 (pyridoxine), B9 (folate), and cortisol via SWCNT E_11_/E_22_ transition-derived nIR fluorescence modulation, providing high biocompatibility (73% adaptability) for precision medicine applications. Its monolithic fabrication supports integration with diverse CoPhMoRe probes, advancing noninvasive diagnostics and multivariate sweat–blood correlation analysis. Song et al. [56] proposed a 3D-printed epifluidic elastic electronic skin (e3-skin). The SSE-based additive manufacturing platform is shown in Figure 10d. The e3-skin integrates MXene-based temperature sensors, CNT-PDMS pressure sensors, and enzyme-functionalized electrochemical biosensors (e.g., GOx/AOx-modified CNT-SBS electrodes with MXene-Prussian blue mediators) to simultaneously monitor sweat glucose, alcohol, pH, and physiological biomarkers. Through iontophoresis-induced sweat stimulation and 3D-printed microfluidics, as detailed in Figure 10e, the system enables autonomous sweat induction and real-time sampling. The integration of MXene micro-supercapacitors (MSCs) interfaced with solar cells facilitates sustainable operation. In human trials, the e3-skin coupled with ridge regression models and SHAP analysis predicted alcohol-induced behavioral impairments (reaction time and inhibitory control) with >90% accuracy. The sensor detects alcohol-induced behavioral disorders by monitoring changes in the concentration of specific biomarkers in real time and correlating the state of central nervous system depression. This fully 3D-printed semisolid extrusion (SSE)-fabricated platform demonstrates customizable wearable systems for personalized remote healthcare monitoring.

## 4. Biomechanical Sensors

Mechanical sensors can be classified into four major categories based on their working principles: piezoresistive [57,58], capacitive [59,60], piezoelectric [61,62], and optical [63]. Piezoresistive sensors detect force signals through the resistance changes caused by material deformation and are suitable for static pressure measurement; capacitive sensors are based on the capacitance changes resulting from the alteration of electrode spacing, featuring high sensitivity and dynamic response characteristics; piezoelectric sensors utilize the charge output characteristics of piezoelectric materials and are adept at high-frequency dynamic force detection; optical sensors convert mechanical signals through modulation of light intensity, wavelength or phase, and have outstanding resistance to electromagnetic interference. Traditional sensors have limitations in terms of adaptability to complex mechanical environments—characterized by high-frequency dynamics, multi-factor coupling, and intense interactions during operation, such as high humidity, pressure fluctuations, and multi-directional force loads—as well as limitations in multi-modal perception and flexible integration. Bionic sensors, however, have achieved breakthroughs in sensitivity, environmental adaptability, and functional integration by imitating the mechanical perception mechanisms of living organisms. For example, multi-layer flexible sensors that imitate human skin can simultaneously detect pressure, shear force and vibration; bionic hair sensors inspired by insect tentacles can achieve high-resolution perception of air flow and contact force. This type of sensor shows broad prospects in soft robots, intelligent prosthetics and wearable medical devices, and will develop in the direction of multi-functional integration, self-healing and intelligence in the future.

The core difficulties faced by biomechanical sensors include signal transmission distortion caused by material–tissue interface mismatch, multidimensional force signal coupling interference in complex environments (such as pressure and bending signal confusion), and insufficient mechanical stability and biocompatibility for long-term use. These problems may be solved through these solutions: drawing on the gradient characteristics of biological tissues (such as skin), constructing asymmetric Janus structure sensitive layers (such as MXene polyurethane composite films), achieving selective response to different mechanical signals through material phase separation, and effectively distinguishing pressure, bending, and tensile signals; combining machine learning techniques such as dynamic Bayesian networks and convolutional neural networks to decouple multi-source signals in real-time and enhance anti-interference capabilities; utilizing dynamic bonding (such as hydrogen bonding/covalent bonding) materials to achieve self-healing of sensitive layer damage, while optimizing packaging processes (such as microfluidic integration) to enhance environmental stability.

**Figure 11:**
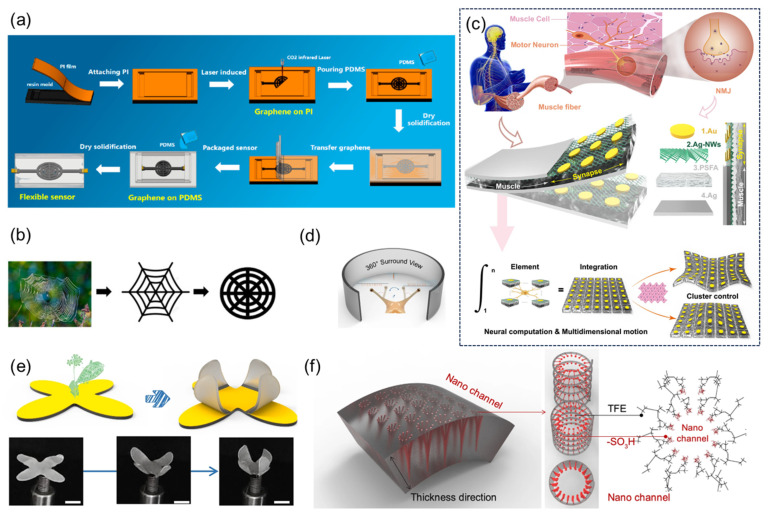
(**a**) Flowchart of the manufacturing process of the flexible sensor with spider web structure. Reproduced with permission from ref. [64] with permission from MDPI. (**b**) From natural spider webs to improved spider web structures. Reproduced with permission from ref. [64] with permission from MDPI. (**c**) Schematic diagram of biological neuromuscular, schematic diagram of biomimetic neuromuscular junction, known as synaptic-motor coupler device (SMCD), which consists of four parts: Au, Ag nanowires (AG-NWS), perfluorosulfonic acid ionomer (PFSA), and Ag. Reproduced with permission from ref. [65] with permission from Springer. (**d**) 360° panoramic schematic diagram, inspired by the eyepiece of a snail. Reproduced with permission from ref. [65] with permission from Springer. (**e**) Schematic diagram of the soft robot design inspired by the Venus flytrap and optical photos of the deformation process, scale: 1 cm. Reproduced with permission from ref. [65] with permission from Springer. (**f**) Schematic magnification of the chemical structure of the nanochannels. Reproduced with permission from ref. [65] with permission from Springer.

Zheng et al. [64] systematically verified the performance advantages of the spider web-structured flexible sensor through laser-induced graphene preparation and bionic structure design. The research team innovatively designed the bionic spider web topology structure (Figure 11a,b) and constructed the radial–spiral composite network through laser direct writing technology. Mechanical simulation shows that this structure can evenly distribute local stress to the whole. The experiment was conducted using a universal testing machine in combination with an LCR meter for quantitative testing: within the strain range of 0–35%, the sensor sensitivity coefficient reached 36.8. Dynamic cyclic tests indicated that after 3000 cycles of 20% strain loading, the deviation of the resistance signal was less than 10%, demonstrating the stability of the structure.

Liu et al. [65] developed a flexible synaptic-motor coupler device (SMCD) that integrates neuromorphic computation and mechanical actuation functionalities (Figure 11c), overcoming the physical separation limitations inherent in conventional systems. This device employs a perfluorosulfonic acid ionomer (PFSA)-based nanochannel network, modified with polyvinyl alcohol (PVA) to form dense hydrophilic nanochannels with a pore size of 2.1 nm. By incorporating a silver nanowire (Ag-NW) forest structure that captures and stores hydrated cations, the device demonstrates synaptic plasticity behaviors including paired-pulse facilitation (PPF), spike-rate-dependent plasticity (SRDP), and spike-number-dependent plasticity (SNDP). It achieves advanced neural functionalities such as Morse code recognition through short-term plasticity, along with nociceptor-mimicking threshold triggering, desensitization, and sensitization. When emulating snail stalk eyes (Figure 11d), the packaged device exhibits 360° panoramic deformation capabilities, maintaining stable operation at a 2 mm bending radius with 10.2 mm displacement output. Through patterned electrode design, multidimensional free shape-morphing control has been implemented in bio-inspired systems including Venus flytrap robots, autonomous hazard detection–avoidance platforms, and multimodal starfish-shaped soft robotics (Figure 11e,f). This technology provides an integrated framework for next-generation neuromorphic robots, edge intelligence devices, and bioelectronic systems.

**Figure 12:**
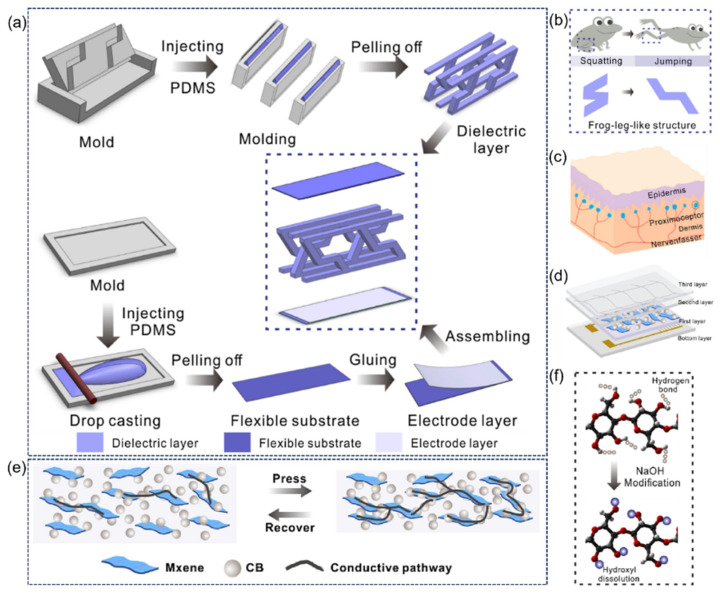
(**a**) The 3D printing fabrication process of 3D biomimetic arrayed frog-leg structure composites and the assembly process of flexible capacitive pressure sensor based on 3D biomimetic arrayed frog-leg structure composites. Reproduced with permission from ref. [66] with permission from Elsevier. (**b**) The two-stage stair structure inspired by frog-leg structure. Reproduced with permission from ref. [66] with permission from Elsevier. (**c**) Schematic diagram of human touch sensation. Reproduced with permission from ref. [67] with permission from Elsevier. (**d**) The design of touch sensation-inspired piezoresistive flexible sensor. Reproduced with permission from ref. [67] with permission from Elsevier. (**e**) The synergistic conductive effect of CB/MXene/SR nanocomposites. Reproduced with permission from ref. [67] with permission from Elsevier. (**f**) Mechanism of utilizing NaOH to modify paper fiber. Reproduced with permission from ref. [67] with permission from Elsevier.

Inspired by the frog leg structure, Zhao et al. [66] proposed a flexible capacitive pressure sensor based on a three-dimensional bionic frog leg structure composite material (the assembly process is shown in Figure 12a). The dielectric layer with a stepped inclined cube array was prepared by 3D printing technology to simulate the layered pressure-bearing characteristics of frog legs (Figure 12b), effectively reducing the Young’s modulus of the material and improving the structural stability. The optimized sensor exhibits ultra-high performance: the sensitivity is 0.583 kPa^−1^ in the range of 0–1.2 kPa, the minimum detection limit is 0.5 Pa, the response/recovery time is only 40 ms and 45 ms, and the detection range is as wide as 200 kPa. This bionic design realizes the efficient conversion of capacitive signals through the three-dimensional structural deformation of the dielectric layer, and has been successfully applied to human motion monitoring and manipulator grabbing control, providing innovative solutions for wearable electronic devices and intelligent robot perception systems.

Inspired by the multi-layer tactile perception mechanism of human skin, Guo et al. [67] developed a new type of flexible piezoresistive sensor. A three-layer functional structure was constructed through bionic design: a waterproof and breathable membrane simulated the epidermal protective layer, a Modal/PDMS fiber composite simulated the dermal tactile receptor, and a CB/MXene/SR nanocomposite simulated the electromechanical transduction function (Figure 12c–f). The sensor uses a conductive nanocomposite with a corrugated microstructure to achieve an ultra-wide linear detection range of 0.1 to 1700 kPa and a high sensitivity of 2.18 kPa^−1^. It also has a fast response of 100 ms and a long-term stability of >5000 cycles. The bionic structure has been successfully applied to electronic skin, manipulator grip force modulation, and human motion monitoring, providing innovative solutions for wearable devices and extreme pressure monitoring, and demonstrating its application potential in human–computer interaction and healthcare.

**Figure 13:**
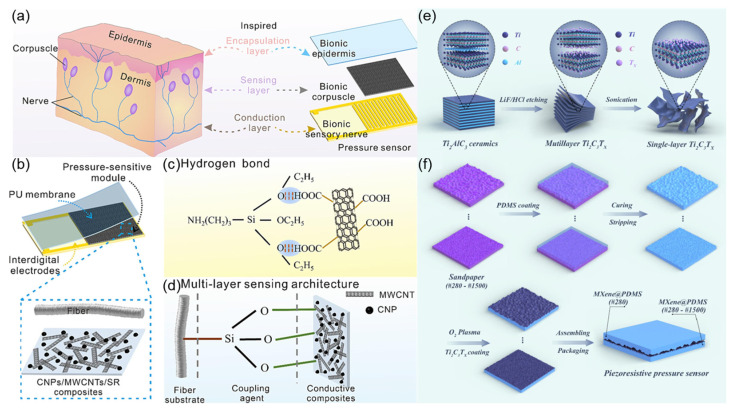
(**a**) Schematic diagram of tactile sensing in the skin and tactile corpuscle-inspired bionic piezoresistive sensor schematic design. Reproduced with permission from ref. [68] with permission from Elsevier. (**b**) Microstructure diagram of the fabric sensing layer. Reproduced with permission from ref. [68] with permission from Elsevier. (**c**) Hydrogen bond interaction between modified modal cellulosic fiber and nanocomposites. Reproduced with permission from ref. [68] with permission from Elsevier. (**d**) Multi-layer sensing architecture of fiber substrate-coupling agent-conductive composites. Reproduced with permission from ref. [68] with permission from Elsevier. (**e**) Schematic illustration of the preparation of Ti3C2Tx (MXene). Reproduced with permission from ref. [69] with permission from Elsevier. (**f**) Schematic illustration of the preparation of MXene@PDMS piezoresistive pressure sensors. Reproduced with permission from ref. [69] with permission from Elsevier.

Inspired by the structure of human skin mechanoreceptors (Figure 13a), Guo et al. [68] designed a bionic sandwich-structured piezoresistive sensor (Figure 13b). By mimicking the layered structure of the epidermis, dermis, and subcutaneous tissue of the skin, the sensor consists of a polyurethane film (bionic epidermis), a pressure-sensitive module (bionic mechanoreceptor), and interdigitated electrodes (bionic sensory nerve). Using APTES coupling agent to modify cellulose fibers (Figure 13c,d), the hydrogen bonding between the conductive nanocomposite and the substrate is enhanced, forming a stable conductive network. The sensor exhibits high sensitivity of 1.0005 kP^−1^, a wide linear detection range of 1700 kPa, and a fast response of 40 ms. This sensor is capable of effectively monitoring human joint movements, gait, and touch operations, with potential applications in wearable electronic skin and smart textiles. This bionic design strategy provides new insights for developing high-performance flexible sensors. Inspired by the microstructure of human skin (Figure 13e), this work [69] constructed a flexible piezoresistive pressure sensor with micro-protruding structures using a sandpaper template method, namely MXene@PDMS. The preparation process is illustrated in Figure 13f. The sensor leverages the excellent conductivity of MXene nanosheets (Figure 13f) and the stress concentration effect of the micro-protruding structures, achieving a high sensitivity of 2.6 kPa^−1^, a fast response/recovery time of 40 ms, and a wide linear detection range of 0–30 kPa. The bionic design effectively enhances the sensitivity to changes in contact area, enabling the sensor to accurately detect physiological signals such as human pulse, joint movement, and vocal cord vibration, and has been successfully applied to handwriting recognition and spatial pressure distribution visualization. This preparation method does not require complex equipment and has the potential for large-scale production, providing a high-performance sensing solution for wearable electronics and smart robotics.

## 5. Multimodal Integrated Sensors

A multimodal sensor is an intelligent device that integrates multiple sensing functions, enabling synchronous acquisition of multi-dimensional signals (temperature, pressure, images, and sounds), as well as precise environmental perception and collaborative analysis through data fusion technology. In contrast, as extensively analyzed in [70], traditional single modal sensors exhibit fundamental limitations. Conventional heart rate monitors based on photoplethysmography (PPG) or electrocardiogram (ECG) principles are restricted to unidimensional data collection, failing to capture hormonal or metabolic changes in complex conditions such as thyroid dysfunction. Similarly, enzymatic glucose sensors measure blood sugar but cannot monitor related physiological parameters like interstitial fluid pH and electrolyte concentrations (e.g., K^+^/Na^+^), significantly limiting personalized diabetes management. Their structurally inflexible designs, susceptibility to motion artifacts, and poor wearing comfort further lead to inaccurate results in scenarios like sleep monitoring.

Multimodal sensor systems, particularly electronic skin (e-skin), offer promising solutions to these limitations, with their bio-inspired designs potentially expanding the boundaries of sensory capabilities [71]. E-skin is a biomimetic technology that mimics human skin’s multimodal sensing capabilities via a flexible, stretchable network of integrated sensors. By combining pressure, temperature, strain, and optical sensors with carbon nanotubes and elastomers, it replicates tactile, thermal, and hair-like sensing mechanisms. State-of-the-art research focuses on flexible substrates, optimized integration architectures, and anti-interference algorithms (e.g., adaptive noise cancellation), achieving synchronous multi-physical detection through biomimetic designs such as vertical stacked layers and three-dimensional (3D) tactile arrays. E-skin shows remarkable potential in robotic tactile interaction and wearable health monitoring: hair-inspired microstructures enable hydrodynamic sensing (e.g., flow velocity), while neuromorphic machine learning (e.g., spiking neural networks) facilitates texture recognition [71]. Despite challenges in material stability and high-density integration, advances in self-healing materials and low-power designs are driving e-skin toward bio-inspired systems [72]. Inspired by natural cilia, e-skin development has accelerated progress in manufacturing techniques and material science. Its ability to process multidimensional data and adapt to dynamic environments highlights the transformative advantage of multimodal integration over traditional single-modal sensors’ limited functionality and poor adaptability.

## 6. Outlook

This article reviews the current state of bionic sensors from four aspects: bioelectric signal sensors, biomarker sensors, biomechanical sensors, and multimodal sensors. Bionic sensors have been successfully applied in various real-world scenarios. Table 1 below introduces biomimetic sites and corresponding sensor types. This field is rapidly advancing and will undoubtedly continue to influence human social and living environments. Meanwhile, bionic sensors still face limitations in certain tasks. For instance, due to material response speed constraints, they struggle to detect high-frequency vibrations. In addition, biomimetic materials such as hydrogels may be affected by high temperatures or corrosive environments, compromising detection accuracy.

In the current era of rapid technological development, intelligent bionic sensors, as the core components for information collection, are profoundly influencing the progress of various fields. As a discipline that integrates traditional wisdom with modern innovation, bionics provides a continuous source of inspiration for breakthroughs in intelligent sensors. By simulating the sophisticated perception mechanisms of living organisms, intelligent sensors have achieved qualitative leaps in sensitivity, multi-functionality, and environmental adaptability, continuously expanding the boundaries of human cognition about the world. Looking to the future, the research and development of bionic sensors should not only continue to seek innovative inspiration from nature but also comprehensively consider key factors such as ecological friendliness, the ethical boundaries of biomimetic intelligence, and the scientific reliability of technological implementation.

To enhance the performance and availability of bionic sensors in physiological activity monitoring and bioinformatics parameter detection, several challenges need to be addressed. In practical application scenarios, significant individual variations in human organs and skin, as well as the complex mechanical properties and behaviors of biomechanics at both macroscopic and microscopic levels, pose rigorous tests for the accuracy and reliability of these sensors. Moreover, the intricate composition of bodily fluid samples further complicates detection. For the monitoring of biological information, bionic sensor arrays can be developed by combining sensing technologies of different principles and the design of signal conversion units. Such sensor arrays are expected to improve the sensitivity and specificity. Bionic sensors also hold potentials for miniaturization, high throughput, and intelligence. These sensors can be integrated with microfluidic systems, chip laboratories and compact circuit systems, paving the way for portable monitoring systems and wearable analysis platforms. Furthermore, large-scale verification of bionic sensors requires the support of clinical applications.

Ethical principles for wearable and implantable bionic devices, including safety and reliability, for example, implant materials such as conductive hydrogels need to pass long-term biocompatibility verification to ensure stable operation in a dynamic environment; and fair accessibility, avoiding cost escalation due to precious metal electrodes or nanomaterials, and ensuring the right to use for vulnerable groups. The emergence of new recognition devices for biological signals such as brain patterns indicates that we need to pay attention to the confidentiality of biological signals. In research or clinical applications, data need to be desensitized to prevent the leakage of personal privacy; at the same time, it is required to have appropriate dynamic supervision, update the review methods and content with the upgrading of technology.

In conclusion, bionic sensors have broad application prospects in scenarios such as life sciences, smart healthcare, and medical diagnostics. Technological progress mainly focuses on miniaturization, long-term continuous monitoring, and improvements in sensitivity, specificity, and biocompatibility. This will contribute to the development of more effective, accurate, and practical applications of bionic sensors in healthcare, home care, clinical diagnosis, treatment optimization, and prognosis assessment. Addressing manufacturing challenges to achieve industrialized production of biomimetic tactile electronic skins, and exploring whether their performance can surpass biological prototypes, remain critical research directions [72]. Improving the efficiency of signal [73] or image processing algorithms and ensuring long-term stable in vivo and in vitro signal acquisition are topics worthy of in-depth research. In future studies, how to apply more biological structures and functions from nature to bionic sensors also needs to be further explored [74]. Through interdisciplinary collaboration among engineers, scientists, and medical professionals, bionic sensors show great potential in advancing health management and improving quality of life [75,76].

## Figures and Tables

**Table 1:** Comparison table of biological models and simulation techniques.

Organism	Simulation Section	Biomimetic Features	Sensor Type
Ref. [10] Octopus	Sucker	Negative pressure adsorption mechanism	ECG sensor
Ref. [13] Starfish	Five-radiation symmetric structure	Flexible adhesive interface	Multimodal cardiac monitoring sensor
Ref. [55] Gecko tree frog	Foot	Mechanical decoupling design reduces motion interference	Biomimetic breathable patch
Ref. [47] Ginkgo	Vein	Nano-pits	Sweat biomarker sensor
Ref. [65] Snail	Snail stalk eye	Wedge-shaped microchannel-oriented sweating	Neuromorphic coupling device
Ref. [67] Frog	Legs	Capillary force-driven	Capacitive pressure sensor
Ref. [68] Human	Skin tactile corpuscle	Multi-layer structure (epidermis-dermis-subcutaneous), corrugated microstructure stress concentration	Piezoresistive pressure sensor
Ref. [20] Human	Scalp curvature	Elastic modulus matching	EEG sensor

## Data Availability

Data are contained within the article.

## References

[1] Xue J., Zou Y., Deng Y., Li Z. (2022). Bioinspired Sensor System for Health Care and Human-Machine Interaction. EcoMat.

[2] Wang Z., Wu Y., Niu Q. (2020). Multi-Sensor Fusion in Automated Driving: A Survey. IEEE Access.

[3] Calvini R., Pigani L. (2022). Toward the Development of Combined Artificial Sensing Systems for Food Quality Evaluation: A Review on the Application of Data Fusion of Electronic Noses, Electronic Tongues and Electronic Eyes. Sensors.

[4] He Y., Xu X., Xiao S., Wu J., Zhou P., Chen L., Liu H. (2024). Research Progress and Application of Multimodal Flexible Sensors for Electronic Skin. ACS Sens..

[5] Yuan Z., Shen G. (2023). Materials and Device Architecture towards a Multimodal Electronic Skin. Mater. Today.

[6] Chen H., Liao L., Zhao X., Fan H., Zhang H., Ren K., Jia D. (2023). High-Sensitivity Electrocardiogram Sensor Based on Fano Resonance in a Double-Stub-Assisted Plasmonic Micro-Ring Resonator. Opt. Laser Technol..

[7] Ullas Pradhan U., Reddy N., Chandrashekar K., Mohan C.B. (2021). Titanium Dioxide Based Bioelectric Sensor for the Acquisition of Electrocardiogram Signals. Microchem. J..

[8] Gromer M., Salb D., Walzer T., Madrid N.M., Seepold R. (2019). ECG Sensor for Detection of Driver’s Drowsiness. Procedia Comput. Sci..

[9] Yasin M., Zulkarnaen M., Yhuwana Y.G.Y., Zaidan A.H., Harun S.W. (2019). Bundled Plastic Optical Fiber Based Sensor for ECG Signal Detection. Optik.

[10] Lian Z., Wang L., Jiang Y., Zhang S., Liu Y., Yu D., Wang W. (2025). 3D-Printed Octopus-Inspired PAM/CS Hydrogels with Excellent Adhesion for High-Performance ECG Sensors. Chem. Eng. J..

[11] Nan X., Mei S., Xu L., Chai J., Wu G., Zhang X., Wang X., Zhao Y., Lv F., Gao L. (2025). Wearable ECG Signal Sensing System Based on Easily Peelable Highly Conductive Hydrogel. Sens. Actuators A Phys..

[12] Zhang Q., Ji K., Huo T., Khan M.N., Hu Z., Yuan C., Zhao J., Chen J., Wang Z., Dai Z. (2022). Biomimetic Patch with Wicking-Breathable and Multi-Mechanism Adhesion for Bioelectrical Signal Monitoring. ACS Appl. Mater. Interfaces.

[13] Chen S., Ouyang Q., Meng X., Yang Y., Li C., Miao X., Chen Z., Zhao G., Lei Y., Ghanem B. (2025). Starfish-Inspired Wearable Bioelectronic Systems for Physiological Signal Monitoring during Motion and Real-Time Heart Disease Diagnosis. Sci. Adv..

[14] He H., Chen H., Huang Z., Zhang J., Zhou N., Zhang H., Fan H., Jia D. (2024). High-Sensitivity Nanostructure-Based Sensor Using Fano Resonance for Noninvasive EEG Monitoring. Measurement.

[15] Jakab K., Csipor J., Ulbert I., Keresztes Z., Mészáros G., Márton G. (2022). EEG Sensor System Development Consisting of Solid Polyvinyl Alcohol–Glycerol–NaCl Contact Gel and 3D-Printed, Silver-Coated Polylactic Acid Electrode for Potential Brain–Computer Interface Use. Mater. Today Chem..

[16] da Silva Souto C.F., Pätzold W., Paul M., Debener S., Wolf K.I. (2022). Pre-Gelled Electrode Grid for Self-Applied EEG Sleep Monitoring at Home. Front. Neurosci..

[17] Yang H., Qian Z., Wang J., Feng J., Tang C., Wang L., Guo Y., Liu Z., Yang Y., Zhang K. (2022). Carbon Nanotube Array-Based Flexible Multifunctional Electrodes to Record Electrophysiology and Ions on the Cerebral Cortex in Real Time. Adv. Funct. Mater..

[18] O’Leary G., Groppe D.M., Valiante T.A., Verma N., Genov R. (2018). NURIP: Neural Interface Processor for Brain-State Classification and Programmable-Waveform Neurostimulation. IEEE J. Solid-State Circuits.

[19] Mlandu N., McCormick S.A., Davel L., Zieff M.R., Bradford L., Herr D., Jacobs C.A., Khumalo A., Knipe C., Madi Z. (2024). Evaluating a Novel High-Density EEG Sensor Net Structure for Improving Inclusivity in Infants with Curly or Tightly Coiled Hair. Dev. Cogn. Neurosci..

[20] Motiepor K., Bakhtiyari S., Jahanshahi A., Bagherzadeh R. (2025). Stretchable Clutter-Free E-Textile EEG Cap: Advancing Seamless Wearable Solutions for Emerging Neuroimaging Applications. Sens. Actuators A Phys..

[21] Liang Q., Xia X., Sun X., Yu D., Huang X., Han G., Mugo S.M., Chen W., Zhang Q. (2022). Highly Stretchable Hydrogels as Wearable and Implantable Sensors for Recording Physiological and Brain Neural Signals. Adv. Sci..

[22] Ge X., Guo Y., Gong C., Han R., Feng J., Ji J., Sun Z., Gao J., Bian F., Xu Z. (2023). High-Conductivity, Low-Impedance, and High-Biological-Adaptability Ionic Conductive Hydrogels for Ear-EEG Acquisition. ACS Appl. Polym. Mater..

[23] Dogan D., Acarman T. (2025). Assessment of Driver Situation for Control Authority Transition from Conditionally Automated Vehicles Using Chassis and Galvanic Skin Response Sensors. Procedia Comput. Sci..

[24] Song M.-S., Kang S.-G., Lee K.-T., Kim J. (2019). Wireless, Skin-Mountable EMG Sensor for Human–Machine Interface Application. Micromachines.

[25] Xu H., Xiong A. (2021). Advances and Disturbances in SEMG-Based Intentions and Movements Recognition: A Review. IEEE Sens. J..

[26] Dai Y., Wu J., Fan Y., Wang J., Niu J., Gu F., Shen S. (2022). MSEva: A Musculoskeletal Rehabilitation Evaluation System Based on EMG Signals. ACM Trans. Sens. Netw..

[27] Bao T., Xie S.Q., Yang P., Zhou P., Zhang Z. (2022). Towards Robust, Adaptive and Reliable Upper-Limb Motion Estimation Using Machine Learning and Deep Learning--A Survey in Myoelectric Control. IEEE J. Biomed. Health Inform..

[28] AL-Quraishi M.S., Elamvazuthi I., Tang T.B., AL-Qurishi M., Parasuraman S., Borboni A. (2021). Multimodal Fusion Approach Based on EEG and EMG Signals for Lower Limb Movement Recognition. IEEE Sens. J..

[29] Shi S., Ming Y., Wu H., Zhi C., Yang L., Meng S., Si Y., Wang D., Fei B., Hu J. (2023). A Bionic Skin for Health Management: Excellent Breathability, in Situ Sensing, and Big Data Analysis. Adv. Mater..

[30] Tian H., Li X., Wei Y., Ji S., Yang Q., Gou G.-Y., Wang X., Wu F., Jian J., Guo H. (2022). Bioinspired Dual-Channel Speech Recognition Using Graphene-Based Electromyographic and Mechanical Sensors. Cell Rep. Phys. Sci..

[31] Huang Q., Chen J., Zhao Y., Huang J., Liu H. (2024). Advancements in Electrochemical Glucose Sensors. Talanta.

[32] Shen H., Shi Y., Zhao P., Wu H., Chen Y., Yang W., Zhu T., Zhang W., Chen X. (2025). A Novel Glucose Sensor Based on the Cu2O/RGO Decorated SWCNT Buckypaper as a Flexible Electrode. Microchem. J..

[33] Sunstrum F.N., Khan J.U., Li N.-W., Welsh A.W. (2025). Wearable Textile Sensors for Continuous Glucose Monitoring. Biosens. Bioelectron..

[34] Ren J., Li Q., Feng K., Gong J., Li Z., Liu X., Yang L., Zhang J. (2025). A Wearable Sensor Based on Janus Fabric upon an Electrochemical Analysis Platform for Sweat Glucose Detection. Talanta.

[35] Chen Y., Sun Y., Li Y., Wen Z., Peng X., He Y., Hou Y., Fan J., Zang G., Zhang Y. (2024). A Wearable Non-Enzymatic Sensor for Continuous Monitoring of Glucose in Human Sweat. Talanta.

[36] Qin J., Jiang S., Wang Z., Cheng X., Li B., Shi Y., Tsai D.P., Liu A.Q., Huang W., Zhu W. (2022). Metasurface Micro/Nano-Optical Sensors: Principles and Applications. ACS Nano.

[37] Vatsal A., Pandey A.K. Zinc Oxide and MXene Based Plasmonic Sensor for Glucose Monitoring. Proceedings of the 2022 Workshop on Recent Advances in Photonics (WRAP).

[38] Yu R., Pan C., Chen J., Zhu G., Wang Z.L. (2013). Enhanced Performance of a ZnO Nanowire-Based Self-Powered Glucose Sensor by Piezotronic Effect. Adv. Funct. Mater..

[39] Gao J., Meng T., Lu S., Ma X., Zhang Y., Fu D., Lu Z., Li C.M. (2020). Manganese-Doped Tremella-like Nickel Oxide as Biomimetic Sensors toward Highly Sensitive Detection of Glucose in Human Serum. J. Electroanal. Chem..

[40] Zou L., Wang S., Qiu J. (2020). Preparation and Properties of a Glucose Biosensor Based on an Ionic Liquid-Functionalized Graphene/Carbon Nanotube Composite. New Carbon Mater..

[41] Asadian E., Hekmat F., Hafezi Kahnamouei M., Mohammadpour R., Shahrokhian S., Sasanpour P. (2025). Supercapacitor-Powered Wearable Biosensor for Continuous Lactate Monitoring from Sweat. Biosens. Bioelectron..

[42] He Q., Wang C., Jain R., Byrnes J., Farquhar E.R., Reed E., Berezovsky E., Chance M.R., Lodowski D., An R. (2024). An Engineered Lactate Oxidase Based Electrochemical Sensor for Continuous Detection of Biomarker Lactic Acid in Human Sweat and Serum. Heliyon.

[43] Tao B., Yang W., Miao F., Zang Y., Chu P.K. (2022). A Sensitive Enzyme-Free Electrochemical Sensor Composed of Co_3_O_4_/CuO@MWCNTs Nanocomposites for Detection of L-Lactic Acid in Sweat Solutions. Mater. Sci. Eng. B.

[44] Han D., Li X., Liang Z., Zhao B., Wu Z., Han F., Han D., Niu L. (2023). Label-Free Photoelectric Sensor for Lactic Acid Determination in Human Sweat. Chin. Chem. Lett..

[45] Karkovska M., Smutok O., Stasyuk N., Gonchar M. (2015). L-Lactate-Selective Microbial Sensor Based on Flavocytochrome B2-Enriched Yeast Cells Using Recombinant and Nanotechnology Approaches. Talanta.

[46] Dykstra G., Chapa I., Liu Y. (2024). Reagent-Free Lactate Detection Using Prussian Blue and Electropolymerized-Molecularly Imprinted Polymers-Based Electrochemical Biosensors. ACS Appl. Mater. Interfaces.

[47] Liao C., Li S., Yang C., Du C., Yao H., Han Z., Stachewicz U., Liu Y. (2025). Wearable Epidermal Sensor Patch with Biomimetic Microfluidic Channels for Fast and Time-Sequence Monitoring of Sweat Glucose and Lactate. Talanta.

[48] Fu J., Wang Y., Ding Y., Wang J., Deng S., Jiang Z., Tan C.S., Li S. (2025). Wearable Ring Sensor for Monitoring Biomarkers of Atherosclerosis in Sweat. Talanta.

[49] Guo L., Zhao Y., Huang Q., Huang J., Tao Y., Chen J., Li H.-Y., Liu H. (2024). Electrochemical Protein Biosensors for Disease Marker Detection: Progress and Opportunities. Microsyst. Nanoeng..

[50] Shahub S., Lin K.-C., Muthukumar S., Prasad S. (2022). A Proof-of-Concept Electrochemical Skin Sensor for Simultaneous Measurement of Glial Fibrillary Acidic Protein (GFAP) and Interleukin-6 (IL-6) for Management of Traumatic Brain Injuries. Biosensors.

[51] Rajan A., Vishnu J., Shankar B. (2024). Tear-Based Ocular Wearable Biosensors for Human Health Monitoring. Biosensors.

[52] Akgönüllü S., Kılıç S., Esen C., Denizli A. (2023). Molecularly Imprinted Polymer-Based Sensors for Protein Detection. Polymers.

[53] Ciou S.-H., Hsieh A.-H., Lin Y.-X., Sei J.-L., Govindasamy M., Kuo C.-F., Huang C.-H. (2023). Sensitive Label-Free Detection of the Biomarker Phosphorylated Tau−217 Protein in Alzheimer’s Disease Using a Graphene-Based Solution-Gated Field Effect Transistor. Biosens. Bioelectron..

[54] Siavash Moakhar R., Del Real Mata C., Jalali M., Shafique H., Sanati A., de Vries J., Strauss J., AbdElFatah T., Ghasemi F., McLean M. (2022). A Versatile Biomimic Nanotemplating Fluidic Assay for Multiplex Quantitative Monitoring of Viral Respiratory Infections and Immune Responses in Saliva and Blood. Adv. Sci..

[55] Song Y., Tay R.Y., Li J., Xu C., Min J., Shirzaei Sani E., Kim G., Heng W., Kim I., Gao W. (2023). 3D-Printed Epifluidic Electronic Skin for Machine Learning–Powered Multimodal Health Surveillance. Sci. Adv..

[56] Lee Y.S., Shin S., Kang G.R., Lee S., Kim D.W., Park S., Cho Y., Lim D., Jeon S.H., Cho S.-Y. (2025). Spatiotemporal Molecular Tracing of Ultralow-Volume Biofluids via a Soft Skin-Adaptive Optical Monolithic Patch Sensor. Nat. Commun..

[57] Gidts M., Hsu W.-F., Payo M.R., Kushwaha S., Wang C., Ceyssens F., Reynaerts D., Locquet J.-P., Kraft M. A Novel Piezoresistive Pressure Sensor Based on Cr-Doped v2O3 Thin Film. Proceedings of the 2023 IEEE 36th International Conference on Micro Electro Mechanical Systems (MEMS).

[58] Pan H., Chen G., Chen Y., Di Carlo A., Mayer M.A., Shen S., Chen C., Li W., Subramaniam S., Huang H. (2022). Biodegradable Cotton Fiber-Based Piezoresistive Textiles for Wearable Biomonitoring. Biosens. Bioelectron..

[59] Wang P., Li J., Yu W., Li G., Meng C., Guo S. (2022). Flexible, Stretchable, Breathable and Sweatproof All-Nanofiber Iontronic Tactile Sensor for Continuous and Comfortable Knee Joint Motion Monitoring. Nano Energy.

[60] Teramoto A., Iba K., Murahashi Y., Shoji H., Hirota K., Kawai M., Ikeda Y., Imamura R., Kamiya T., Watanabe K. (2021). Quantitative Evaluation of Ankle Instability Using a Capacitance-Type Strain Sensor. Foot Ankle Int..

[61] De Fazio R., Del-Valle-Soto C., Mastronardi V.M., De Vittorio M., Visconti P. (2024). A Smart Glove to Evaluate Parkinson’s Disease by Flexible Piezoelectric and Inertial Sensors. Sens. Int..

[62] Zhang M., Tan Z., Zhang Q., Shen Y., Mao X., Wei L., Sun R., Zhou F., Liu C. (2023). Flexible Self-Powered Friction Piezoelectric Sensor Based on Structured PVDF-Based Composite Nanofiber Membranes. ACS Appl. Mater. Interfaces.

[63] Large M.C.J., Moran J., Ye L. (2009). The Role of Viscoelastic Properties in Strain Testing Using Microstructured Polymer Optical Fibres (MPOF). Meas. Sci. Technol..

[64] Zheng H., Wang H., Yi K., Lin J., Chen A., Chen L., Zou Z., Liu M., Ji Y., Dong L. (2023). Wearable LIG Flexible Stress Sensor Based on Spider Web Bionic Structure. Coatings.

[65] Liu J., Jiang C., Yu Q., Ni Y., Yu C., Xu W. (2025). Multidimensional Free Shape-Morphing Flexible Neuromorphic Devices with Regulation at Arbitrary Points. Nat. Commun..

[66] Zhao Y., Guo X., Hong W., Zhu T., Zhang T., Yan Z., Zhu K., Wang J., Zheng G., Mao S. (2023). Biologically Imitated Capacitive Flexible Sensor with Ultrahigh Sensitivity and Ultralow Detection Limit Based on Frog Leg Structure Composites via 3D Printing. Compos. Sci. Technol..

[67] Guo X., Hong W., Hu B., Zhang T., Jin C., Yao X., Li H., Yan Z., Jiao Z., Wang M. (2023). Human Touch Sensation-Inspired, Ultrawide-Sensing-Range, and High-Robustness Flexible Piezoresistive Sensor Based on CB/MXene/SR/Fiber Nanocomposites for Wearable Electronics. Compos. Struct..

[68] Guo X., Zhang T., Wang Z., Zhang H., Yan Z., Li X., Hong W., Zhang A., Qian Z., Zhang X. (2024). Tactile Corpuscle-Inspired Piezoresistive Sensors Based on (3-Aminopropyl) Triethoxysilane-Enhanced CNPs/Carboxylated MWCNTs/Cellulosic Fiber Composites for Textile Electronics. J. Colloid Interface Sci..

[69] Chen B., Zhang L., Li H., Lai X., Zeng X. (2022). Skin-Inspired Flexible and High-Performance MXene@Polydimethylsiloxane Piezoresistive Pressure Sensor for Human Motion Detection. J. Colloid Interface Sci..

[70] Guzman P., Dinh T., Qamar A., Lee J., Zheng X.Q., Feng P., Rais-Zadeh M., Phan H.-P., Nguyen T., Md Foisal A.R. (2022). Thermal-piezoresistive Pumping on Double SiC Layer Resonator for Effective Quality Factor Tuning. Sens. Actuators A Phys..

[71] Jeon S., Lim S.-C., Trung T.Q., Jung M., Lee N.-E. (2019). Flexible Multimodal Sensors for Electronic Skin: Principle, Materials, Device, Array Architecture, and Data Acquisition Method. Proc. IEEE.

[72] Yu J., Ai M., Liu C., Bi H., Wu X., Ying W.B., Yu Z. (2025). Cilia-Inspired Bionic Tactile E-Skin: Structure, Fabrication and Applications. Sensors.

[73] Nazari V., Zheng Y.-P. (2023). Controlling Upper Limb Prostheses Using Sonomyography (SMG): A Review. Sensors.

[74] Masalskyi V., Dzedzickis A., Korobiichuk I., Bučinskas V. (2025). Hybrid Mode Sensor Fusion for Accurate Robot Positioning. Sensors.

[75] Zhu Y., Moyle W., Hong M., Aw K. (2025). From Sensors to Care: How Robotic Skin Is Transforming Modern Healthcare—A Mini Review. Sensors.

[76] Davis-Wilson H.C., Maldonado-Rosado E., Hegarty-Craver M., Temple D.S. (2025). Potential for Wearable Sensor-Based Field-Deployable Diagnosis and Monitoring of Mild Traumatic Brain Injury: A Scoping Review. Sensors.

